# Early-onset restrictive food intake disorders in children: a latent class analysis

**DOI:** 10.1007/s00787-023-02316-3

**Published:** 2023-10-27

**Authors:** Coline Stordeur, Anaël Ayrolles, Vincent Trebossen, Ségolène Barret, Florence Baillin, Hélène Poncet-Kalifa, Carine Meslot, Julia Clarke, Anne Bargiacchi, Hugo Peyre, Richard Delorme

**Affiliations:** 1 Child and Adolescent Psychiatry Department, Rare Disease Refence Center for Early-Onset Anorexia Nervosa, Robert Debré University Hospital, APHP, Paris, France; 2 Université Paris Cité, Paris, France; 3 Human Genetics & Cognitive Functions, CNRS UMR3571, Institut Pasteur, Paris, France; 4 Pôle Universitaire de Psychiatrie de l’Enfant et de l’Adolescent (P.U.P.E.A), CH Laborit, Poitiers, France; 5 Institute of Psychiatry and Neuroscience of Paris, INSERM U1266, Paris, France; 6 CMME (GHU Paris Psychiatrie et Neurosciences), Paris Descartes University, Paris, France; 7 Centre de Ressources Autisme Languedoc-Roussillon et Centre d’Excellence Sur l’Autisme et les Troubles Neuro-développementaux, CHU Montpellier, 39 Avenue Charles Flahaut, 34295 Montpellier Cedex 05, France; 8 UMRS 1141, INSERM, Paris, France

**Keywords:** Early-Onset Anorexia Nervosa, ARFID, Diagnosis, LCA

## Abstract

The two most frequent early-onset restrictive food intake disorders are early-onset anorexia nervosa (EOAN) and avoidant/restrictive food intake disorders (ARFID). Although the core symptoms of EOAN (*i.e.*, fear of gaining weight and disturbed body image) are not present in ARFID, these symptoms are difficult to assess during the initial phase of hospitalisation. Our aim was  to identify restrictive food intake disorder subtypes in children using latent class analysis (LCA) based on the information available at admission to hospital, and to determine the agreement between the subtypes identified using LCA and the final diagnosis: EOAN or ARFID. We retrospectively included 97 children under 13 years old with severe eating disorders (DSM-5) at their first hospitalisation in a specialised French paediatric unit. LCA was based on clinical information, growth chart analyses and socio-demographic parameters available at admission. We then compared the probabilities of latent class membership with the diagnosis (EOAN or ARFID) made at the end of the hospitalisation. The most parsimonious LCA model was a 2-class solution. Children diagnosed with EOAN at the end of hospitalisation had a 100% probability of belonging to class 1 while children diagnosed with ARFID had an 8% probability of belonging to class 1 based on parameters available at admission. Our results indicate that clinical and socio-demographic characteristics other than the core symptoms of EOAN may be discriminating for a differential diagnosis.

**Supplementary Information:**

The online version contains supplementary material available at 10.1007/s00787-023-02316-3.

## Introduction

Early-onset restrictive food intake disorders represent a heterogeneous group of conditions. In the DSM-5, the two most frequent restrictive food intake disorders in children are avoidant/restrictive food intake disorders (ARFID) and early-onset anorexia nervosa (EOAN) (American Psychiatric Association 2013). EOAN and ARFID are both characterised by severe food restriction and weight loss and mainly differ with a fear of gaining weight and a distorted body image in EOAN but not in ARFID [3, 8]. Unlike anorexia nervosa (AN), where food restriction is driven by a desire to control weight and by a distorted body image, food avoidance in ARFID is driven more by a lack of interest in eating, the sensory characteristics of food, or a fear of the physical consequences of eating (*e.g.* choking, vomiting, or a fear of somatic issues)(American Psychiatric Association 2013). In a latent class analysis (LCA), Pinhas et al. [16] found that a 2-class solution of core symptoms describing restrictive food intake disorder (in particular, fear of gaining weight and body image disturbance) closely resemble the criteria for EOAN and ARFID with similar results in Australian, Canadian and British children. Similar finding have been replicated underlying the importance of considering shape concern as a key differentiator to distinguish between different phenotypes of children with food restrictions [17].

In clinical practice, the differential diagnosis between EOAN and ARFID is difficult during the initial stages of care in children with a restrictive food intake disorder, body image disturbances are less informative in children than in adults with AN, as only half of children with EOAN felt their body was larger than it is [8, 15]. Thus it could be useful to identify other markers of each disorder because they each require specific treatment and prognosis. Moreover, a reliable clinical assessment of the core symptoms of restrictive food intake disorder (*i.e.*, fear of gaining weight and body image disturbance) can take several weeks. Although the initial phases of hospital treatment for acute malnutrition may be comparable in EOAN and ARFID, other treatments (such as family-based therapy, individual therapy, and medication for comorbidities) are highly dependent on a precise diagnosis of the restrictive food intake disorder [5, 8]. This can sometimes result in a late diagnostic re-evaluation which may affect the prognosis in the absence of adapted management.

Some studies have compared the clinical characteristics of children with ARFID and EOAN. A higher prevalence of males (60% in ARFID *versus* 6% in EOAN), an earlier onset of illness (6 *versus* 13 years), a longer time to diagnosis, and more frequent abdominal pain are reported in children with ARFID when compared with those with EOAN [3, 6]. These studies also found a higher prevalence of psychiatric comorbidities including Attention Deficit Hyperactivity Disorder (ADHD), autism spectrum disorder (ASD) and anxiety disorders in children with ARFID as compared to those with EOAN [6, 12, 13, 18]. On the contrary, the mood disorders were more prevalent in children with EOAN than ARFID [7, 14]. In their latent class analysis (LCA), Pinhas et al*.* found that patients with EOAN were older and with more weight loss, while patients with ARFID were more likely to have a comorbid psychiatric disorder [16]. Despite these differences, both disorders can have somatic consequences/complications requiring inpatient treatment.

To our knowledge, there are no studies that have evaluated the precise diagnostic value of clinical and socio-demographic characteristics available at hospital admission in determining the final diagnosis in children with food intake disorder without including core symptoms of AN in the analysis. To fill that gap, we aim to identify subtypes of restrictive food intake disorder in children using latent class analysis (LCA) only based on the clinical information, growth chart analyses and socio-demographic parameters available at admission to hospital. We then evaluated the agreement between the subtypes identified with LCA and the final diagnosis at the end of the hospitalisation (EOAN or ARFID).

## Method

### Participants and measures

Children from 8 to 12 years old with first hospital admission at the Child and Adolescent Psychiatry Department, Robert Debré University Hospital (Paris, France) from January 2018 to 2020 with restrictive food intake disorder were included (*N* = 97).

Initial inpatient clinical assessments were carried out by a psychiatrist specialised in children eating disorder in charge of the hospitalisation for each patient according to a standardised clinical assessment framework. Then, socio-demographic and clinical data were retrospectively collected from hospital admission reports (including socio-demographic data, sports habits, age at onset, age at hospitalisation, personal and family medical history, clinical characteristics at hospitalisation admission and growth chart analysis). Eating-disorder diagnosis and comorbid psychiatric diagnosis (anxiety disorders, depressive disorders, and neurodevelopmental disorders) were determined using DSM-5 criteria. For accuracy, growth chart analyses were performed by two psychiatrists specialised in children’s eating disorders and with an expertise in nutrition. We also collected the patient diagnosis (EOAN or ARFID) at the end of hospitalisation.

## Statistical analysis

LCA was performed to identify subtypes of restrictive food intake disorder [10].We examined models with 1 to 4 classes to determine the model with the optimal number of classes. Twenty-one elements of clinical information, growth chart analyses and socio-demographic parameters available at of admission to hospital were selected. The 21 variables used for LCA were: sex, Tanner stage, socio-economic level (high socio-economic level was define as at least one parent with executive and higher intellectual professions), age at first symptoms, intensive sports activity (over 4 h a week of extracurricular sport), first degree eating disorder history (including AN, bulimia nervosa, but also reported selective and restrictive eating difficulties in first degree relatives even if the diagnosis of ARFID was not established), first degree psychiatric history, developmental delay (included walking delay, speech and language delay and intellectual delay), feeding disorder during the first year, personal chronic disease history, reported trigger for the onset of the eating disorder (as teasing, critical comments or even harassment with or without a link to weight or physical appearance), psychiatric comorbidities, generalised anxiety disorder, obsessional compulsive disorder (OCD), major depressive disorder (MDD), ASD, specific learning disorder, ADHD, growth retardation, early adiposity rebound, absence of adiposity rebound. The variables chosen for LCA were based on an expert’s appraisal and previous evidence in the literature [2, 16, 19].

Fit indices (including Akaike’s Information Criterion (AIC) and Bayesian Information Criterion (BIC)) were assessed to determine the best fit model, with lower values indicating better fitted models with the appropriate number of classes [11]. We then compared probability of class membership with diagnosis reported for patients at the end of hospitalisation (EOAN versus ARFID). We also provided as supplementary information a group comparison between EOAN and ARFID participants with the Fisher’s exact test and controlling for the false discovery rate for the 21 variables previously used in LCA model as supplementary. Analyses were performed with RStudio version 1.3.959.

### Ethics statement

This study was performed under the recommendations of the local ethics committee (Comité de l’Evaluation de l’Ethique des projets de Recherche de Robert Debré, CEER-RD n°2021-520ter).

## Results

**Fig. 1:**
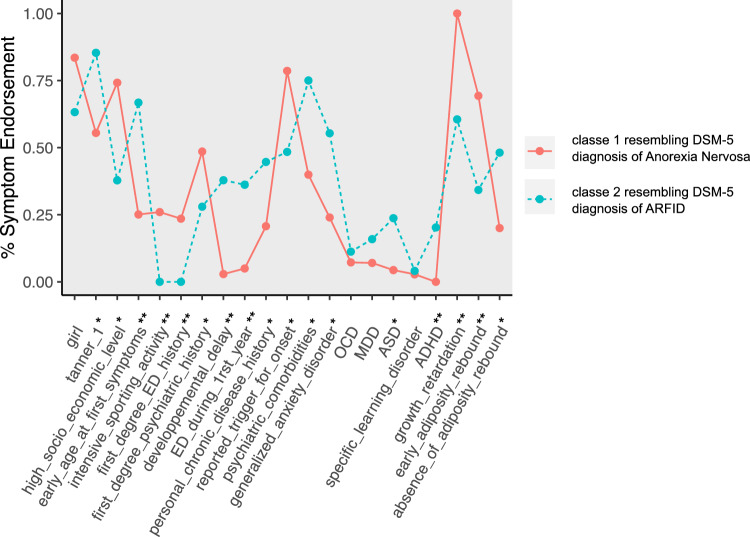
Differences in eating disorder symptom endorsement between the two clusters identified by latent class analysis. Cluster 1, solid line; Cluster 2 dashed line. *p < 0.05; **p < 0.0

A total of 97 children were included in the study, the sample was predominantly female (78%) with a mean age of 11.30 years and a sex-adjusted BMI percentile of 11 at admission. The mean age at first reported eating or feeding disorder symptoms was 9.50 years. LCA was performed on the total sample (*n* = 97). The most parsimonious LCA model was a 2-class solution based on the AIC and BIC fit indices (Table 1). Cluster 1 had more girls, a higher socioeconomic level, more intensive sports activity and an earlier Tanner stage than cluster 2. Moreover, patients in cluster 1 more frequently had an history of eating disorders and other psychiatric history in first degree relatives but fewer personal histories of developmental delay, chronic disease, or feeding disorders during first year of life, as well as fewer personal psychiatric comorbidities (including generalised anxiety disorder, MDD, ASD, ADHD) compared to cluster 2. The probabilities of having OCD and specific learning disorders were similar in clusters 1 and 2. A specific trigger was more frequently found for the disease onset in cluster 1 and these patients presented with early adiposity rebound and growth retardation at admission. There was a lower proportion of adiposity rebound in cluster 2 than in cluster 1 (Fig. 1; Table 2).

**Table 1:** Testing latent class analyses models for most parsimonious model

Model	BIC score	AIC	LL	*Df*
2 clusters	1889	1776	– 844	53
3 clusters	1962	1790	– 828	30
4 clusters	1953	1721	– 770	7

Two-cluster model was most parsimonious

*LL* log-likelihood; *df* degrees of freedom

**Table 2:** Comparisons between Cluster 1 (resembling DSM-5 diagnosis of Anorexia Nervosa) and Cluster 2 (resembling DSM-5 criteria for Avoidant/Restrictive Food Intake Disorder) (*n* = 97)

	Cluster 1 (*n* = 72)	Cluster 2 (*n* = 25)	*p* values
Girls	0.84	0.63	0.05
Tanner 1	0.55	0.85	**0.03**
High socio-economic level	0.74	0.38	** < 0.01**
Early age at first symptoms	0.25	0.67	** < 0.001**
Intensive sporting activity	0.26	0	** < 0.01**
First degree ED history	0.24	0	** < 0.01**
First degree psychiatric history	0.49	0.28	0.10
Developmental delay	0.03	0.38	** < 0.001**
Eating or feeding disorder during first year	0.05	0.36	** < 0.001**
Personal chronic disease history	0.21	0.45	**0.04**
Reported trigger for ED onset	0.79	0.48	** < 0.01**
Psychiatric comorbidities	0.40	0.75	** < 0.01**
Generalised anxiety disorder	0.24	0.55	** < 0.01**
OCD	0.07	0.11	0.42
MDD	0.07	0.16	0.23
ASD	0.04	0.24	** < 0.01**
Specific learning disorder	0.03	0.04	1
ADHD	0.00	0.20	** < 0.001**
Growth retardation	1.00	0.60	** < 0.001**
Early adiposity rebound	0.69	0.34	**0.01**
Absence of adiposity rebound	0.20	0.48	**0.02**

*ED* eating disorder; *OCD* Obsessive compulsive disorder; *MDD* major depressive disorder; *ASD* Autism spectrum disorder; *ADHD* attention deficit hyperactivity disorder. Bold values denote statistical significance at the p<0.05 level.

**Fig. 2:**
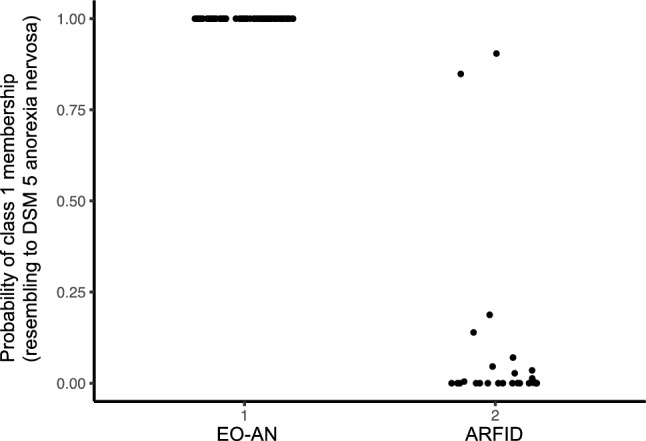
The probability of class 1 membership (resembling to DSM 5 anorexia nervosa diagnosis) according to the initial diagnosis

Cluster 1 included 74% (*n* = 72) of the sample (based on the most likely class membership) and exhibited characteristics resembling a diagnosis of EOAN as described in Herpertz − Dahlmann et al*.* [8]. Cluster 2 was distinct from the EOAN group and resembled to the DSM-5 category for ARFID. Thus, the probability of class 1 membership according to the initial diagnosis was 100% for EOAN patients and 8% for ARFID patients (Fig. 2).

There were 2 diagnostic misclassifications (based on the most probable class membership) with LCA model with 2 ARFID patients categorised in cluster 1 resembling to a DSM-5 diagnosis of AN).

We also provided a comparison between EOAN (*n* = 70) and ARFID (*n* = 27) patients based on diagnosis at hospitalisation discharge. Differences in the group comparison (Supp Table S1) were similar to those in the cluster comparison (Table 2) with the presence of more comorbid psychiatric and neurodevelopmental disorders (70% vs 41%, *p* = 0.03) and a higher likelihood of a history of chronic disease (45% vs 20%, *p* = 0.04) in ARFID group, as well as an earlier age at first reported eating or feeding disorder symptoms (*p* =  < 0.001). Furthermore, although these differences were not statistically significant, there was a higher proportion of males in ARFID group (37% vs 16%, *p* = 0.06) as well as more frequent onset from Tanner stage 1(63% vs 44%, *p* = 0.06). Based on auxological parameters, children with EOAN had more frequent early adiposity rebound (50% vs 33%, *p* = 0.02) than those with ARFID while the adiposity rebound did not occur in more children with ARFID (41% vs 17%, *p* = 0.07). We also reported growth retardation in 95% of EOAN patients and in 34% of ARFID patients at hospitalisation admission.

## Discussion

The present study identified two subtypes of restrictive food intake disorder in children using LCA based on clinical information, growth chart analyses and sociodemographic parameters available at hospital admission. The clusters found with LCA based on the parameters at admission were consistent with the diagnosis of EOAN and ARFID made at the end of hospitalisation. Compared to previous LCA studies in children with restrictive eating disorders [16, 17], we did not include core symptoms of anorexia nervosa (i.e., fear of gaining weight and disturbed body image) in our variables because these symptoms are often difficult to identify in children at the beginning of treatment. We demonstrated that clinical and sociodemographic characteristics other than the core symptoms of EOAN available at admission to hospital can help obtain a highly accurate diagnosis of children with food intake disorder (only two ARFID patients were misclassified in EOAN group).

Cluster 1, representing 74% of the sample, was mostly associated with a diagnosis of EOAN at the end of hospitalisation. Patients in cluster 1 were more often girls, with a higher socioeconomic level, intensive sports activity, more frequent familial eating disorders other psychiatric histories and more frequently had an identified trigger for the onset of their eating disorder. Although these features are consistent with the classic features of EOAN [8], it should be noted that they are not always present in single patients. In our sample, none of the patients from cluster 1 had associated ADHD but all had a retarded growth curve. These features must be interpreted as a whole to determine the diagnosis. Cluster 2 represented 26% of the sample, had features suggesting a diagnosis of ARFID [7] and was associated with a diagnosis of ARFID at the end of hospitalisation.

Differences between ARFID and EOAN groups were consistent with previous studies and helped characterise patients with ARFID (see Supp Table S1) with the presence of earlier onset of symptoms and more comorbid psychiatric and neurodevelopmental disorders and a higher likelihood of a history of chronic disease [3, 6, 7, 14, 15]. Unlike one previous observational study, we found that children with EOAN more frequently had first-degree psychiatric histories (including a history of ED) than those with ARFID (Kurotori et al., 2019). This difference may be explained by different samples size in the two studies (13 ARFID patients in Kurotori study versus 27 in ours).

To our knowledge, this is the only study that has compared auxological parameters in children with ARFID and those with EOAN. We reported growth retardation in 34% of ARFID patients at hospitalisation, although this was less frequent than in children with EOAN (95%, *p*** < **0.001), it is to be noted that undernutrition can also lead to growth impairment in ARFID patients. In ARFID, the health consequences of reduced food intake may be similar to those in EOAN (hypothermia, anaemia, bradycardia, tooth decay) [15] thus, early and intensive treatment to restore weight is indicated [3].

Accurate diagnosis of children with food intake disorder (ARFID or EOAN) at early time at hospitalisation admission is helpful to define treatment strategy and adequate psychoeducation for patients and families. ARFID is a heterogeneous group that includes various subtypes: chronic low appetite, or limited interest in feeding, fear of aversive consequences (fear of choking or vomiting), severe selective eating and/or food neophobia. A single patient may belong to more than one subtype due to frequent overlap of eviction drivers [4]. In our hospitalisation sample, reported primary drivers of food avoidance for ARFID patients were mainly fear of aversive consequences (48%), low appetite/food interest (30%) and sensory sensitivity (22%). It is particularly useful to identify additional factors that differentiate ARFID patients with acute food restriction from EOAN patients with similar symptoms. Refeeding strategies, dietary diversification targets or weight targets differ between ARFID and EOAN, early accurate diagnosis helps providing adequate and individualised treatment, especially relevant psychotherapy, and allows the healthcare team to better tailor their attitude and treatment goals to the patient.

Our results may be interpreted in the light of several limits. As for many retrospective studies, the reliability of the initial clinical data assessment by different psychiatrist can be questioned, to address this issue, initial inpatient clinical assessments was carried out according to a standardised clinical assessment framework for all patient and retrospective collection was carried out in a standardised manner by trained psychiatrists specialised in eating disorders. Additionally, the appropriate sample size in LCA is not clearly defined, large sample sizes above 250 participants are preferred, our sample remains limited (*n* = 97) but smaller sample sizes can be adapted in simpler models with larger class differences (Nylund-Gibson & Choi, 2018), our 2 cluster model with clear differences between the two group appear to be reliable. Finally, this study was not designed to explore the heterogeneity of ARFID, more specific question related to ARFID presentations has been recently addressed in larger sample size, confirming a 3 distinct clusters in LCA corresponding to the 3 ARFID subtypes described in the literature, and emphasizing the need for individualised treatment [9].

## Conclusion

Our results support the presence of two distinct diagnoses of early-onset restrictive food intake disorder in children. Moreover, our results identified additional reliable features to guide early diagnosis and inpatient treatment criteria in patients with severe eating disorders when core symptoms of anorexia nervosa (i.e., fear of gaining weight and body image disturbance) are not found. Further studies are needed to evaluate whether early identification of the precise diagnosis of restrictive food intake disorder improves the clinical outcome of children.

### Supplementary Information

Below is the link to the electronic supplementary material.

Supplementary file1 (DOCX 23 KB)

## Data Availability

De-identified individual participant data (including data dictionaries) will be made available, in addition to study protocols, the statistical analysis plan, and the informed consent forms. The data will be made available upon publication to researchers who provide a methodologically sound proposal for use in accordance with the agreements of the ethics committee. Proposals should be submitted to coline.stordeur@aphp.fr.
