# Supplementary material for: Early-onset restrictive food intake disorders in children: a latent class analysis
Source: Eur Child Adolesc Psychiatry. 2023 Oct 27;33(7):2273–9. doi: 10.1007/s00787-023-02316-3 (PMC11255037; doi:10.1007/s00787-023-02316-3)
Supplement: Supplementary file 1 — Supplementary file1 (DOCX 23 KB) [file 787_2023_2316_MOESM1_ESM.docx]

**Early-onset restrictive food intake disorders in children:**

**A latent class analysis.**

**SUPPLEMENTARY**

**TABLE S1** Group comparison between participants with Early-Onset Anorexia Nervosa (EOAN) and Avoidant/Restrictive Food Intake Disorder (ARFID) (n=97)

|  | EOAN  (n=70) | ARFID  (n=27) | p values with FDR correction |
| --- | --- | --- | --- |
| Girls | 0.84 | 0.63 | 0.06 |
| Tanner 1 | 0.44 | 0.63 | 0.06 |
| High socio-economic level | 0.63 | 0.37 | **0.04** |
| Early age at first symptoms | 0.25 | 0.60 | **<0.01** |
| Intensive sporting activity | 0.26 | 0 | **<0.001** |
| First degree ED history | 0.24 | 0 | **<0.01** |
| First degree psychiatric history | 0.48 | 0.29 | **0.03** |
| Developmental delay | 0.03 | 0.33 | **<0.001** |
| Eating or feeding disorder during first year | 0.05 | 0.30 | **<0.001** |
| Personal chronic disease history | 0.20 | 0.45 | **0.04** |
| Reported trigger for ED onset | 0.77 | 0.52 | **0.03** |
| Psychiatric comorbidities | 0.41 | 0.70 | **0.03** |
| Generalized anxiety disorder | 0.24 | 0.52 | **0.03** |
| OCD | 0.07 | 0.11 | 0.83 |
| MDD | 0.07 | 0.15 | 0.38 |
| ASD | 0.04 | 0.22 | **0.03** |
| Specific learning disorder | 0.03 | 0.04 | 1 |
| ADHD | 0.00 | 0.18 | **<0.01** |
| Growth retardation | 0.95 | 0.34 | **<0.001** |
| Early adiposity rebound | 0.50 | 0.33 | **0.02** |
| Absence of adiposity rebound | 0.17 | 0.41 | 0.07 |
| *ED: Eating disorder; OCD: Obsessive compulsive disorder; MDD: Major depressive disorder; ASD: Autism spectrum disorder; ADHD: Attention deficit hyperactivity disorder; FDR: False discovery risk* | | | |
